# Patient satisfaction with antiretroviral services at primary health-care facilities in the Free State, South Africa – a two-year study using four waves of cross-sectional data

**DOI:** 10.1186/1472-6963-8-210

**Published:** 2008-10-09

**Authors:** Edwin Wouters, Christo Heunis, Dingie van Rensburg, Herman Meulemans

**Affiliations:** 1 Department of Sociology and Research Centre for Longitudinal and Life Course Studies, University of Antwerp, Sint-Jacob Street 2, 2000 Antwerp, Belgium; 2 Centre for Health Systems Research & Development, University of the Free State, Nelson Mandela Avenue, Bloemfontein, South Africa

## Abstract

**Background:**

The study's first objective was to determine the levels of patient satisfaction with services at antiretroviral treatment (ART) assessment sites. Differences in patient satisfaction with several aspects of service over time and among health districts were measured. The second objective was to examine the association between human resource shortages and levels of patient satisfaction with services.

**Methods:**

Four cross-sectional waves of data were collected from a random sample of 975 patients enrolled in the Free State's public-sector ART programme. One-way analysis of variance (ANOVA) with the Bonferroni adjustment for multiple comparisons was used to assess the differences in patient satisfaction among the Province's five districts and among the four waves of data. Correlation coefficient analysis using Pearson's *r *was used to assess the association between ART nurse vacancy rates and patient satisfaction with the services provided by nurses over time.

**Results:**

With respect to both general services and the services provided by nurses, our results indicate high overall satisfaction among Free State patients receiving public-sector ART. However, our data present a less positive picture of patient satisfaction with waiting times. Patients in Fezile Dabi District were generally slightly dissatisfied with the waiting times at their assessment sites. In fact, waiting times at assessment sites were the most important predictor of discontent among ART patients. Significant geographical (*P *< 0.001) and temporal differences (*P *< 0.005) were observed in these three aspects of patient satisfaction. Patients were most satisfied in Thabo Mofutsanyana District and least satisfied in Motheo District. Patients in Fezile Dabi District were generally slightly dissatisfied with the waiting times at their assessment sites. Finally, our analysis revealed a strong negative association (*r *= -0.438, *P *< 0.001) between nurse vacancy rates and mean satisfaction levels with services performed by nurses at baseline. Patients attending facilities with high professional nurse vacancy rates reported significantly less satisfaction with nurses' services than did those attending facilities with fewer vacant nursing posts.

**Conclusion:**

Collectively, our findings show high levels of patient satisfaction with ART-related services, but also confirm claims by other studies, which have identified human resource shortages as the most important obstacle to a successful South African AIDS strategy.

## Background

An estimated 5.5 million South Africans are currently infected with HIV, making South Africa's HIV/AIDS epidemic one of the worst in the world [1]. To address this epidemic, South Africa has introduced antiretroviral (ARV) treatment (ART) through the *Operational Plan for Comprehensive HIV and AIDS Care, Management and Treatment *in late 2003 (hereinafter the *Comprehensive Plan*). The *Comprehensive Plan *specifies primary health-care (PHC) clinics and community health centres as the main sites for the diagnosis, staging, and routine follow-up of HIV-positive patients [2]. These assessment sites are specially equipped, nurse-driven PHC facilities that serve as entry points to the public ART programme. Here, patients are screened and staged by professional ART-trained nurses. Subsequently, eligible patients are referred to treatment sites for follow-up tests. After a doctor has certified the patient for ART, the assessment site also becomes the point of drug-readiness training, monthly drug issue, and PHC delivery [3].

As has been observed in many industrialized countries, the provision of ART via the public health system can transform AIDS from a fast, insidious killer into a more manageable, though still incurable, chronic illness [4-9]. However, in resource-limited settings, there are many challenges in successfully scaling-up ART and reorienting service delivery towards chronic disease care. Although the still-significant gap in funding is acknowledged, shortages in human resources for health care are often cited as the most important obstacle to a successful treatment scale-up [10-12]. HIV/AIDS further fuels the absolute shortages of health workers in sub-Saharan and southern Africa [13-17]. Together, the large numbers of eligible patients and the labour-intensive public-sector ART programme overstretch the health system and overburden health staff [11,12,18]. This is also the case in South Africa and the Free State Province [3,19].

Weakened and overloaded health systems threaten the quality of care and patient satisfaction levels, which can, in turn, seriously lessen the chances of a successful AIDS strategy. The importance of the link between human resource shortages and patient responsiveness to the ART programme, defined as the extent to which health systems meet patients' expectations of how they should be treated, has already been noted by Schneider at al. (2006) [10]. In a weakened health system, it is even more crucial to ensure a high quality of care and patient satisfaction, to maximize the benefits of the scarce resources. In the past decade in particular, patient satisfaction has become an important performance measure and outcome of health care [20-22]. An understanding of how patients evaluate their care may help to identify deficiencies and inform improvements, to render health-care programmes more patient-centred and to increase their efficiency in a context of scarce resources [23]. However, research examining patient satisfaction with health care provision in South Africa is limited [24-27]. Moreover, patients' views on quality of public-sector ARV care are relatively unexplored [25,26].

In accordance with the directives of the *Comprehensive Plan*, the Free State Province opted for a strong PHC-oriented ART scale-up. Because the Free State's strategy depends heavily on nurse-driven assessment sites, patient satisfaction with the services at these sites could seriously affect the successful implementation of the much-needed ART scale-up. Satisfied patients are more likely to comply with their treatment, which is in turn associated with better clinical outcomes [28-33]. Research is required into the factors that will optimize patient satisfaction with ART-related services and that produce the associated health benefits.

Although high levels of patient satisfaction are important for a successful strategy against HIV/AIDS, research into patient satisfaction with health services in general, and with ART services specifically, has been limited in South Africa [25]. The first objective of this study was to extend on the current literature by examining patient satisfaction levels with ART assessment sites in four cross-sectional waves of interviews with public-sector ART patients in the Free State. In practical terms, we examined differences in patient satisfaction with several aspects of ART-related services, both over time and also across health districts. This study's second objective was to examine the association between human resource shortages and patient satisfaction levels with the services at the assessment sites, measured in the same sample of patients enrolled in the Free State public-sector ART programme.

## Methods

### Sampling

The study population for each of the four cross-sectional interview surveys included all patients 18 years and older enrolled in the public-sector ART programme in the Free State. The population included both those patients already receiving ART and those not yet receiving ART but already certified by a designated doctor as qualifying for treatment, who were required to attend drug-readiness training. The sampling frame consisted of a list of names of patients in each of the five health districts, obtained from the provincial Department of Health. These were adult patients who were medically certified as ready for ART. This sampling frame excluded patients with CD4 counts below 200 cells/μL and those who had reached WHO Stage IV AIDS, but were not certified as ready to commence treatment, often because the patients were first required to complete two months of tuberculosis treatment.

In each wave, a stratified random sample of patients was selected from each of the five districts, and from all those PHC facilities providing ART. During the first wave, 80 patients were randomly sampled per district, proportional to the numbers of patients receiving ART at the 16 PHC facilities and per treatment group (receiving ART or awaiting ART) [34]. In one district with fewer than 80 patients, a census of all the patients receiving treatment or awaiting treatment (certified as qualifying for treatment) was conducted. Because this study aimed to compare patient satisfaction levels over time, new samples of patients were drawn at approximately 6, 12, and 18 months after the first wave, following a similar sampling procedure. In the context of the available resources, the maximum number of new treatment cases sampled in each district in any one period was 40, which we believe still allowed us to generalize to the district and provincial levels in terms of the ART programme. The total sample size was 975 patients (Table 1).

**Table 1:** Patient numbers by wave and health district (N = 975)

		**Survey wave**				**Total**
		**Wave 1**	**Wave 2**	**Wave 3**	**Wave 4**	

**Health District**	Lejweleputswa	81	44	39	39	203
	Motheo	81	44	36	41	202
	Fezile Dabi	84	39	40	39	202
	Thabo Mofutsanyana	81	41	38	38	198
	Xhariep	44	46	40	40	170

**Total**	Free State	371	214	193	197	975

### Data collection

Face-to-face interviews were conducted by trained interviewers in Sotho, Afrikaans, or English. The interviews took place at the 16 assessment sites. Written, informed consent was first obtained from the study participants by the nursing personnel at the assessment sites, and later again by the enumerators. The study was approved by the Ethics Committee of the Faculty of Humanities (University of the Free State). In-field editing of the completed questionnaires took place regularly, with immediate feedback to the interviewers in cases where problems and inconsistencies were encountered.

### Measures

In order to measure patient satisfaction with ART services in the Free State Province of South Africa, we first needed to develop a valid and reliable questionnaire which was easy to complete for HIV/AIDS patients enrolled in the public-sector ART programme. The need to devise and test a new questionnaire measuring patient satisfaction stemmed from the specific nature of the South African public sector ART programme, together with a lack of appropriate tools adequately tested in high-prevalence, resource-limited settings. The items of the questionnaire are the outcome of a literature review and the researchers' visits to the PHC facilities under investigation [25,35,36]. The 12 items selected in our measurement of patient satisfaction at assessment sites in the Free State also resulted from discourses with frontline practitioners in daily contact with the patients under study.

Satisfaction with the assessment site services was measured using 12 five-point Likert-scale items. These covered satisfaction with (i) medical care, (ii) complaint procedure, (iii) cleanliness of the facility, (iv) privacy during examinations, (v) confidentiality of medical records, (vi) respect shown by nurses, (vii) health information about HIV/AIDS, (viii) information provided by nurses on ARV medication, (ix) opportunity to ask questions, (x) language used during consultations, (xi) facility opening hours, and (xii) waiting time before consultation. Exploratory factor analysis was performed to examine the factor structure of the satisfaction scale. The factor scores produced were subjected to reliability analysis. Finally, and most importantly, differences in satisfaction scores among various patient groups were assessed using one-way analysis of variance (ANOVA). Bonferroni adjustments for multiple comparisons were made to assess the differences in patient satisfaction among the five health districts, as well as among the four waves of data. All data analysis was performed with the statistical software package SPSS version 15.0.

Because professional nurses are the main front-line providers of public-sector HIV/AIDS care in South Africa, we used data on the vacancy rates for approved professional nursing posts at the assessment sites as a measure of health staff shortages. The first round of data was taken from a staffing audit conducted in November 2004, which counted the approved, filled, and vacant professional nursing posts at the assessment sites in each health district of the Free State [37]. Because the staffing audit and our Wave 1 interviews were conducted around the same time, these data on the nurse staffing at the assessment sites were used as the baseline indication of health staff shortages at the 16 assessment sites. Approximately two years later, information on approved, filled, and vacant nursing posts at the same 16 assessment sites was retrieved per district from the provincial personnel administrative system. Because the retrieval of the staffing data was conducted at approximately the same time (July 2006) as the Wave 4 patient survey, these data were linked to the last wave of the patient survey.

## Results

### Sample description

The mean age of this sample of people living with HIVAIDS (PLWHA) was 37.5 years (SD = 8.5). Of the 975 ART patients who were interviewed, 68.1% were women. Education levels were relatively low, with only 17.2% having completed secondary education and only 2.5% having some form of tertiary education. Very few patients enrolled in the public-sector ART programme were employed at the time of the interview. The unemployment rate in the sample population was a staggering 83.6%. Approximately half the unemployed patients (47.0%) reported being unable to work due to illness or disability. However, our data suggest that patients enrolled in the ART programme were not the poorest of the poor [38]. The majority of respondents (76.4%) lived in formal dwellings, whereas only 19.3% and 4.3% lived in informal and traditional dwellings, respectively. The mean personal monthly income was ZAR642 (= $67.54 [Exchange rate on 09-12-2008]). Approximately two-thirds of respondents (66.4%) were receiving a social welfare grant at the time of the interview.

### Construction of the satisfaction subscales

#### Exploratory factor analysis

Factor analysis is a way of testing the construct validity of a scale by determining whether the components of the scale measure similar constructs by loading onto the same factors. The amount of variance extracted by a factor is an indication of the homogeneity of the scale [39,40].

In our study, exploratory factor analysis (EFA) using principal axis factoring with varimax rotation, with the eigenvalue greater than 1 criterion, produced two factors (Table 2). Factor loadings ≥ 0.4 were considered significant. Factor one, with an eigenvalue of 4.01, explained 28.7% of the variance in patient satisfaction and comprised nine items measuring patients' *satisfaction with the general services *(Factor 1) at the assessment sites: satisfaction with (i) medical care, (ii) complaint procedure, (iii) cleanliness of the facility, (iv) privacy during examinations, (v) confidentiality of medical records, (vii) health information about HIV/AIDS, (ix) opportunity to ask questions, (x) language used during consultations, and (xi) facility opening hours. The second factor had an eigenvalue of 3.0 and explained 21.1% of the variance. Examination of the factor loadings indicated that two items expressing patients' *satisfaction with ART-related services performed by nurses *(Factor 2) were grouped around this stable second factor, comprised of (vi) respect shown by nurses and (viii) information provided by nurses on ARV medication. Only the item measuring *patient satisfaction with waiting times *did not load on either of the two dimensions revealed by the EFA. Therefore, this item was treated separately in subsequent analyses.

**Table 2:** Exploratory factor analysis of patients' satisfaction with assessment site services: factor loadings and reliability analysis

**Items**	**Factor 1**	**Factor 2**
*Factor 1: Satisfaction with general services*		
Medical care^1^	**0.518**	0.280
Complaint procedure^2^	**0.519**	0.221
Cleanliness of the facility	**0.616**	0.291
Privacy during examinations	**0.730**	0.302
Confidentiality of medical records	**0.706**	0.356
Health information about HIV/AIDS	**0.818**	0.173
Opportunity to ask questions	**0.587**	0.218
Language used during consultations	**0.578**	0.340
Facility opening hours	**0.476**	0.305
*Factor 2: Satisfaction with nurses*		
Respect shown by nurses	0.146	**0.587**
Information provided by nurses on ART	0.205	**0.836**
Waiting time before consultation	0.041	0.123
% Variance explained	28.7	21.1
Cronbach's α	0.862	0.722

^1 ^Referring to general medical care provided at the facility.

^2 ^It is policy in South Africa that clinics should have a formal, clear, structured complaint procedure and should also maintain a register of complaints and how they were addressed.

#### Reliability analysis

The internal consistency of the derived factors was measured by calculating Cronbach's α coefficients for both factors (Table 2). It has been recommended that, for the purpose of group comparisons, Cronbach's α should be 0.7 or above [41,42]. The nine-item factor, general satisfaction with the services at the assessment sites, showed high internal consistency with Cronbach's α of 0.862. The overall internal consistency of the two-item factor expressing patient satisfaction with the services performed by the nurses at the assessment sites was 0.722. Repeating these calculations while excluding one item at a time did not improve Cronbach's α and therefore did not detect any redundant items.

#### Factor scores

Finally, raw item scores were summed and divided by the number of items on the scale to produce two factor scores: satisfaction with general services and satisfaction with the services performed by nurses. For all remaining analyses, the two factor scores, as well as the item measuring patient satisfaction with waiting times, are labelled, as gross representations, satisfaction with *general services*, *nurses*, and *waiting times *at the assessment sites.

### Differences in patient satisfaction across health districts

**Figure 1:**
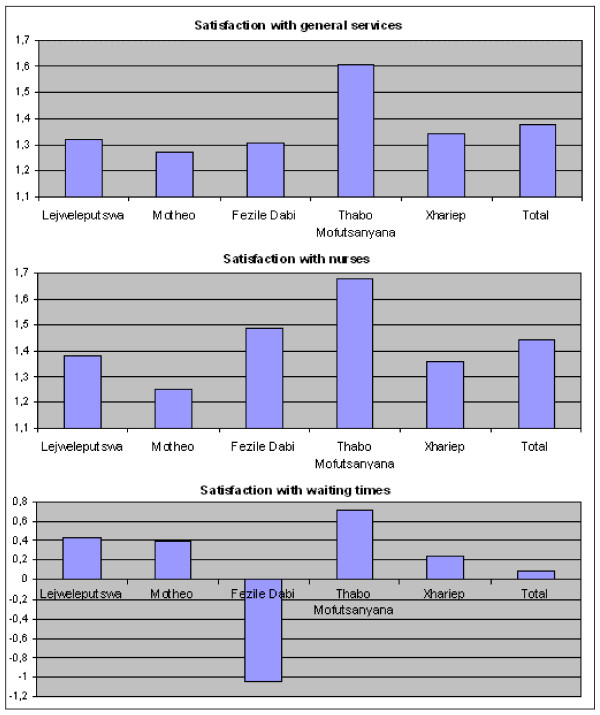
Patient satisfaction by health district (N = 975).

One-way ANOVA was used to assess the association between the health district within which the assessment site was located and the satisfaction levels with its services. Where the results of the ANOVA were statistically significant, post hoc Bonferroni multiple comparisons were made to determine where statistically significant differences between means existed. Values of *P *< 0.05 were considered significant (Figure 1).

First, the data were examined for differences in patient satisfaction across the five health districts. Because satisfaction scores were scaled between -2 (very dissatisfied) and +2 (very satisfied), the overall satisfaction with general services (mean = 1.38, SD = 0.48) and with the services provided by nurses (mean = 1.48, SD = 0.55) was high. Generally, patients were much less satisfied with the waiting times at the assessment sites, because the mean score was barely above the theoretical midpoint of the scale (mean = 0.53, SD = 1.43). A series of one-way ANOVAs revealed significant differences in patient satisfaction with general services (F(4, 792) = 14.9; *P *< 0.001), services performed by nurses (F(4, 960) = 11.5; *P *< 0.001), and waiting times (F(4, 967) = 76.1; *P *< 0.001) among the five health districts (Table 3).

**Table 3:** One-way ANOVA for satisfaction levels with general services, nurses, and waiting times across five health districts (N = 975)

**Variable**	**N**	**Mean**	**SD**	**F**	***P***
Satisfaction with general services	797	1.38	0.48	F(4, 792) = 14.9	< 0.001
Satisfaction with nurses	965	1.49	0.55	F(4, 960) = 11.5	< 0.001
Satisfaction with waiting times	972	0.35	1.43	F(4, 967) = 76.1	< 0.001

To determine exactly where these differences occurred, we examined the results of the Bonferroni multiple comparisons (Table 4). First, regarding patient satisfaction with the services in general, the post hoc analyses revealed that patients from Thabo Mofutsanyana District were significantly more satisfied with the general services than were patients from the four other health districts. These districts did not significantly differ from one another. Second, a post hoc test was conducted to determine how the five districts differed in terms of satisfaction with the services performed by nurses. Again, patients from Thabo Mofutsanyana reported a significantly higher mean satisfaction score than that of patients from the other districts. At the bottom end, patients from Motheo District were significantly less satisfied than were patients from the four other health districts. Satisfaction levels among the three remaining districts did not differ significantly. Finally, multiple comparisons revealed that patients attending assessment sites in Fezile Dabi District were significantly less satisfied with waiting times than were patients in Motheo, Lejweleputswa, Thabo Mofutsanyana, or Xhariep Districts. Furthermore, patients from Thabo Mofutsanyana reported a significantly higher degree of satisfaction with waiting times than did patients in Xhariep. All other differences were not statistically significant.

**Table 4:** Pairwise comparisons: Bonferroni tests for differences in mean satisfaction levels across five health districts (N = 975)

	**Satisfaction with general services**		**Satisfaction with nurses**		**Satisfaction with waiting times**	
**Multiple Comparisons**	**Difference**	***P***	**Difference**	***P***	**Difference**	***P***

L – M	0.048	n.s.	0.130	< 0.05	0.029	n.s.
L – FD	0.014	n.s.	-0.107	n.s.	1.473	n.s.
L – TM	-0.287	< 0.001	-0.295	< 0.001	-0.277	< 0.001
L – X	-0.023	n.s.	0.024	1.000	0.189	n.s.
M – FD	-0.034	n.s.	-0.237	< 0.001	1.444	< 0.001
M – TM	-0.335	< 0.001	-0.425	< 0.001	-0.307	n.s.
M – X	-0.071	n.s.	-0.106	< 0.05	0.160	n.s.
FD – TM	-0.301	< 0.001	-0.188	< 0.005	-1.750	< 0.001
FD – X	-0.037	n.s.	0.131	n.s.	-1.284	< 0.001
TM – X	0.264	< 0.001	0.319	< 0.001	0.467	< 0.05

n.s. not significant (P ≥ 0.05).

L = Lejweleputswa.

M = Motheo.

FD = Fezile Dabi.

TM = Thabo Mofutsanyana.

X = Xhariep.

In summary, patients were most satisfied with services in general at assessment sites in Thabo Mofutsanyana. Patients from Motheo District were least satisfied with the services performed by nurses, and patients from Fezile Dabi were generally very dissatisfied with the waiting times at the assessment sites in their area. Overall, patients rated their assessment site as 'good' or 'excellent'. Satisfaction levels with the general services and the services provided by nurses were generally high, whereas respondents were considerably less satisfied with the waiting times at the assessment sites.

### Changes in patient satisfaction over time

**Figure 2:**
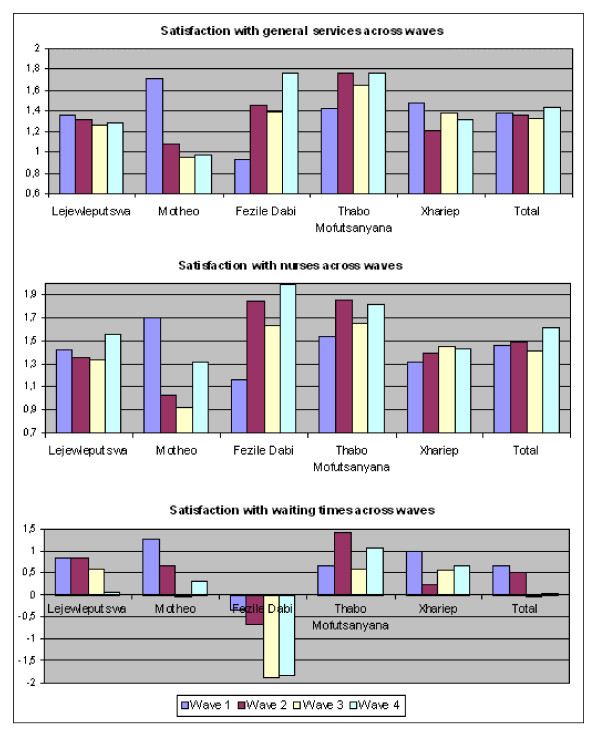
Temporal trends in patient satisfaction by health district (N = 975).

The longitudinal character of the study allowed us to examine trends and changes in patient satisfaction with ART services at the 16 assessment sites over time. Because the previous analysis revealed significant differences in patient satisfaction among the five health districts, we also examined the evolution in patient satisfaction for each district separately. Figure 2 shows an overview of the changes over time in patient satisfaction with general services, services performed by nurses, and waiting times for each district and for the Province as a whole.

First, patient satisfaction with general services did not change significantly over time in Lejweleputswa. However, one-way ANOVA revealed significant changes in patient satisfaction in the four other health districts (Table 5). Satisfaction levels in Motheo declined over subsequent waves (F(3, 165) = 32.3, *P *< 0.001). Multiple comparisons revealed that the decline in patient satisfaction with general services was only significant between Wave 1 and the three other waves (Table 6). This indicates that the satisfaction level decreased between Waves 1 and 2 and that this lower satisfaction level was sustained during subsequent waves. The data for Fezile Dabi revealed a more favourable pattern (F(3, 136) = 27.7, *P *< 0.001). Post hoc tests revealed that patient satisfaction with general services increased between Waves 1 and 3. Furthermore, patient satisfaction in general was significantly higher in Wave 4 than in the three previous waves. In Thabo Mofutsanyana, patient satisfaction with general services increased significantly between Waves 1 and 2. This high satisfaction rate was sustained across Waves 3 and 4. Post hoc tests for Xhariep District indicated that satisfaction with the general services was significantly lower in Wave 2 than at baseline. Multiple comparisons revealed no further significant differences in Xhariep. When we looked at the Free State as a whole, one-way ANOVA revealed no significant differences in patient satisfaction with general services over time.

**Table 5:** One-way ANOVA: temporal trends in patient satisfaction with general services, nurses, and waiting times for each of the five health districts

**Variable**	**N**	**Mean**	**SD**	**F**	***P***
*Satisfaction with general services*					
Lejweleputswa	154	1.32	0.35	F(3, 150) = 0.71	n.s.
Motheo	169	1.27	0.57	F(3, 165) = 32.3	< 0.001
Fezile Dabi	140	1.30	0.54	F(3, 136) = 27.7	< 0.001
Thabo Mofutsanyana	182	1.61	0.40	F(3, 178) = 10.4	< 0.001
Xhariep	152	1.34	0.43	F(3, 148) = 2.7	< 0.05
Total	797	1.38	0.48	F(3, 793) = 1.27	n.s.
*Satisfaction with nurses*					
Lejweleputswa	201	1.41	0.47	F(3, 197) = 1.74	n.s.
Motheo	199	1.34	0.65	F(3, 195) = 21.3	< 0.001
Fezile Dabi	200	1.55	0.57	F(3, 196) = 37.4	< 0.001
Thabo Mofutsanyana	195	1.68	0.49	F(3, 191) = 5.1	< 0.005
Xhariep	170	1.45	0.48	F(3, 166) = 0.40	n.s.
Total	965	1.49	0.55	F(3, 961) = 5.10	< 0.005
*Satisfaction with waiting times*					
Lejweleputswa	203	0.64	1.03	F(3, 199) = 6.36	< 0.001
Motheo	202	0.70	1.26	F(3, 198) = 12.9	< 0.001
Fezile Dabi	201	-1.01	1.43	F(3, 197) = 20.8	< 0.001
Thabo Mofutsanyana	196	0.88	1.29	F(3, 198) = 20.8	< 0.01
Xhariep	170	0.61	1.22	F(3, 166) = 3.2	< 0.05
Total	972	0.35	1.43	F(3, 968) = 14.71	< 0.001

n.s. not significant (P ≥ 0.05)

Second, the temporal trends in patient satisfaction with the services performed by nurses were assessed with ANOVA and post hoc Bonferroni tests. Mean satisfaction with the services performed by nurses did not change significantly in Lejweleputswa or Xhariep. However, temporal effects were observed in the three other health districts (Table 5). In Motheo, Wave 1 respondents reported significantly higher satisfaction levels than those of respondents in subsequent waves. At the end of the study period, the opposite trend was observed in that Wave 4 patients reported significantly higher satisfaction than did Wave 3 patients. However, patient satisfaction was still significantly lower than at baseline. Similar to the changes in satisfaction with general services, the mean satisfaction with services provided by nurses increased significantly in Fezile Dabi. The Wave 1 satisfaction level was significantly lower than that of subsequent waves. At the end of the study period, the mean satisfaction with services provided by nurses had increased significantly for a second time. Finally, mean satisfaction with services by nurses also increased significantly over time in Thabo Mofutsanyana. The Wave 2 level of satisfaction was significantly higher than that at baseline. The small dip in patient satisfaction in Wave 3 was not significant and the mean satisfaction in Wave 4 was again significantly higher than that in Wave 1. In total, Free State patients receiving public-sector ART reported a positive temporal effect on satisfaction with the services provided by nurses (F(3, 961) = 5.1, *P *< 0.005). Compared with Waves 1 and 3, patients reported significantly higher levels of satisfaction in Wave 4 (Table 6).

**Table 6:** Pairwise comparisons: Bonferroni tests for differences in mean satisfaction levels over time for each of the five health districts (only statistically significant differences shown, *P *< 0.05)

	**Satisfaction with general services**		**Satisfaction with nurses**		**Satisfaction with waiting times**	
**Multiple Comparisons**	**Difference**	***P***	**Difference**	***P***	**Difference**	***P***

**Lejweleputswa**						
W1 – W4	-	-	-	-	0.788	< 0.001
W2 – W4	-	-	-	-	0.790	< 0.005
**Motheo**						
W1 – W2	0.633	< 0.001	0.670	< 0.001	0.600	< 0.05
W1 – W3	0.765	< 0.001	0.777	< 0.001	1.315	< 0.001
W1 – W4	0.737	< 0.001	0.391	< 0.005	0.942	< 0.001
W2 – W3	-	-	-	-	0.715	< 0.05
**Fezile Dabi**						
W1 – W2	-0.519	< 0.001	-0.684	< 0.001		
W1 – W3	-0.459	< 0.001	-0.462	< 0.001	1.563	< 0.001
W1 – W4	-0.835	< 0.001	-0.824	< 0.001	1.483	< 0.001
W2 – W3	-	-	-	-	1.208	< 0.001
W2 – W4	-0.316	< 0.05	-	-	1.128	< 0.001
W3 – W4	-0.376	< 0.05	-0.362	< 0.005	-	-
**Thabo Mofutsanyana**						
W1 – W2	-0.352	< 0.001	-0.310	< 0.005	-0.758	< 0.05
W1 – W3	-0.229	< 0.05	-	-	-	-
W1 – W4	-0.340	< 0.001	-0.268	< 0.05	-	-
W2 – W3	-	-	-	-	0.846	< 0.05
**Xhariep**						
W1 – W2	0.266	< 0.05	-	-	0.783	< 0.05

Finally, the most pronounced time effect was observed with respect to satisfaction with waiting times at the assessment sites. One-way ANOVA revealed that there were some significant temporal changes in all five health districts (Table 5).

In Lejweleputswa, Wave 4 participants reported significantly less satisfaction than did those in Waves 1 and 2. Patients in Motheo reported a significant linear decline in satisfaction with waiting times at the assessment sites between Waves 1 and 3. The negative mean value in Wave 3 indicated that patients were generally slightly dissatisfied with the waiting times at their sites. Alarmingly, patients in Fezile Dabi were generally dissatisfied with the waiting times at their sites. Furthermore, they reported a significant decline in satisfaction between Waves 2 and 3, whereas Wave 3 and 4 patients were generally very dissatisfied with waiting times. Post hoc analysis of patient satisfaction in Thabo Mofutsanyana revealed a mixed pattern. Between Waves 1 and 2, patients reported a significant increase in satisfaction with waiting times, but between Waves 2 and 3, this gain was lost, as patients reported a decline in satisfaction compared with the baseline level. In Xhariep, patient satisfaction significantly decreased between Waves 1 and 2. The lower satisfaction level was sustained, and all other differences were non-significant.

Finally, if we consider the differences in patient satisfaction between waves for the entire sample of Free State patients (F(3, 968) = 14.7, P < 0.001), the significant temporal effect on patient satisfaction with waiting times is mainly attributable to a significant reduction in patient satisfaction between Waves 2 and 3 (Table 6).

Generally, the mean satisfaction with the general services and the services provided by nurses underwent a positive evolution in Fezile Dabi and Thabo Mofutsanyana. A negative temporal trend was apparent in Motheo, and to a lesser extent in Xhariep. Patients' declining satisfaction with waiting times was most evident in Fezile Dabi.

### Human resource challenges as a possible explanation

Collectively, our findings not only demonstrate differences in mean patient satisfaction across the five health districts, but also indicate that the evolution in patient satisfaction over time varied across the five health districts. A relevant question for policy-makers would be: What caused these inter-district differences?

**Figure 3:**
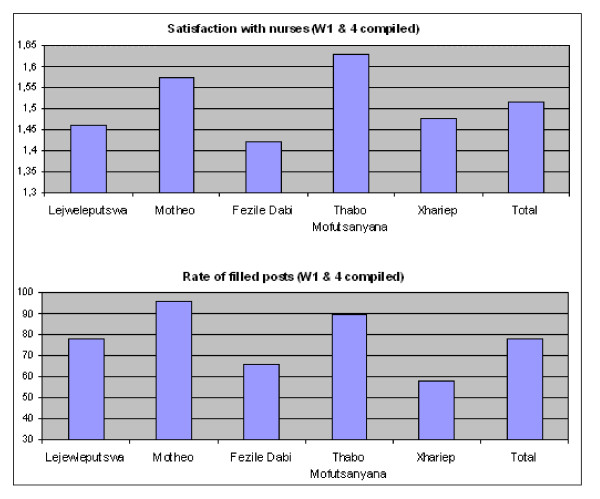
Rates of filled nursing posts and mean satisfaction with nurses by health district (Waves 1 & 4 combined) (N = 568).

First, the analysis described above revealed significant differences in mean patient satisfaction with the services provided by nurses across the five health districts. Because of its prominence in the literature on ART scale-up, health staff shortages (especially shortages in professional nurses in the ART programme) could be a possible explanation for these differences in satisfaction levels. Therefore, in this part of the analysis, we used a correlation procedure (Pearson's *r*) to assess the bivariate relationships between vacant professional nursing posts and patient satisfaction with the services performed by nurses. Only those *r *values for which the two-tailed probability was less than 0.05 were considered statistically significant correlations. A moderate to strong negative correlation (*r *= -0.343, *P *< 0.001) was identified between the rate of vacant nursing posts at the assessment sites per district and the satisfaction level with the services performed by nurses at these sites. Those patients attending an assessment site with relatively few vacant professional nursing posts reported significantly higher levels of satisfaction with the services performed by ART nurses than did patients attending facilities with high vacancy rates. This association is clearly evident when a bar chart showing the mean satisfaction with the services of nurses across the five health districts (Waves 1 and 4 combined) is compared with a bar chart showing the levels of filled ART nursing posts per district (mean vacancy rates for November 2004 and July 2006) (Figure 3). Patients from the relatively well-staffed (with professional nurses) assessment sites of Thabo Mofutsanyana and Motheo reported higher levels of satisfaction than did patients from the three other districts, which show relatively high rates of vacant nursing posts at their ART assessment sites.

**Figure 4:**
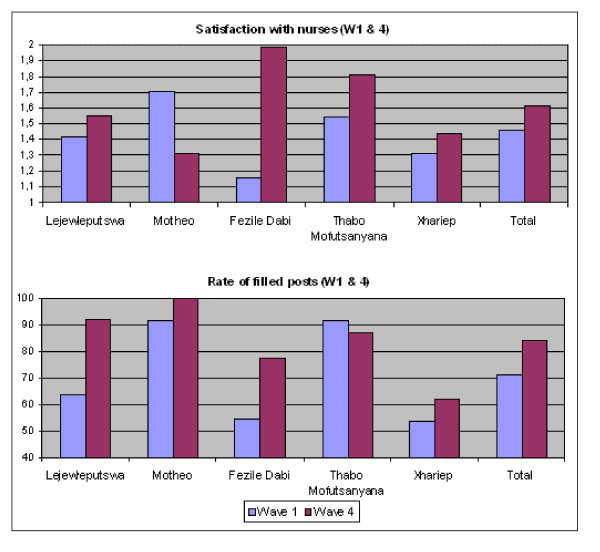
Rates of filled nursing posts and mean satisfaction with nurses by health district (Waves 1 & 4 separately) (N = 568).

Second, in the earlier part of the analysis, ANOVA also indicated that the evolution in patient satisfaction over time varied across the five health districts. Correlation coefficient analysis using Pearson's *r *was also used to assess the association between vacancy rates for ART nurses and patient satisfaction with the services performed by the nurses over time (Table 7). At baseline, a two-tailed probability value of less than 0.001 indicated that the correlation was not the result of either chance or random sampling error. The Pearson *r *value of -0.438 revealed that there was a strong negative association between vacancies in professional nursing posts and mean satisfaction levels with the services provided by nurses at baseline. Patients attending facilities with high levels of professional nurse vacancies (Fezile Dabi and Xhariep) reported significantly lower levels of patient satisfaction with the services performed by nurses than did those in districts with lower vacancy rates (Thabo Mofutsanyana and Motheo) (Figure 4). However, approximately two years later, this negative association was rather weak (*r *= -0.201, *P *< 0.05). At that stage of the programme, the evidence that patient dissatisfaction with ART services could be explained by nursing staff vacancies was weak.

**Table 7:** Bivariate correlations between nurse vacancy rates and satisfaction with ART-related services performed by nurses

**Correlation between**	**Pearson's *r***	**Significance (two-tailed)**
Nurse vacancy rate (W1 & W4) **&**Satisfaction with nurses (W1 & W4)	-0.343	< 0.001
Nurse vacancy rate (W1) **&**Satisfaction with nurses (W1)	-0.438	< 0.001
Nurse vacancy rate (W4) **&**Satisfaction with nurses (W4)	-0.201	< 0.05

## Discussion

The interviews with patients enrolled in the Free State ART programme provided useful insights into the patients' satisfaction with ART-related services at the PHC facilities.

With respect to both the general services and the services provided by nurses, our study indicates high overall satisfaction among Free State ART patients in the public sector. However, significant geographic and temporal differences were observed in the overall satisfaction scores reported by patients. Our results show that the satisfaction levels of patients in the Motheo District declined over time. Conversely, patient satisfaction increased in Fezile Dabi and Thabo Mofutsanyana. There were no clear temporal changes in Xhariep or Lejweleputswa. Although satisfaction levels were generally high, the significant decline in patient satisfaction in Motheo deserves the attention of provincial policy makers and district and facility managers. This study recommends further research into the determinants of these geographic and temporal differences in patient satisfaction.

Our data present a less positive picture of patient satisfaction with waiting times at the assessment sites, especially in Fezile Dabi, where satisfaction with waiting times was not only significantly lower than that in the other four districts, but also declined significantly over time. At baseline, only six of 10 approved professional nursing posts were filled in Fezile Dabi, which undoubtedly contributed to the long waiting times at the assessment sites in this health district. Growing patient numbers and an already-overloaded health system caused waiting times to increase even more sharply. The first wave of another research project of the Centre for Health Systems Research & Development currently in progress, i.e. the FEATS Project, indicates the mean waiting time before consultation at assessment sites in the Free State to be 142 minutes [43]. Overall, dissatisfaction with waiting times at assessment sites seems to have been the most important predictor of discontent among ART patients.

Finally, we also identified a significant inverse correlation between nursing staff shortages and patient satisfaction levels. South Africa's *Comprehensive Plan *opted for a strong PHC-oriented ART scale-up, which depends heavily on professional nurses and necessitates a strengthening of the health system. The staffing requirements for the ART programme stipulated in the *Plan *also indicate that professional nurses are the backbone of the programme. However, the creation of new posts does not necessarily mean they will be filled. Severe health staff shortages (specifically among professional nurses) reduce the chance of the successful implementation of the *Comprehensive Plan*, in that our analyses show that vacancy rates of professional nursing posts at assessment sites were significantly associated with patient satisfaction levels at those sites. Patients attending assessment sites with high vacancy rates in professional ART nursing posts in the districts of Fezile Dabi and Xhariep reported lower levels of satisfaction with ART-related services.

Overall, the results of our analyses argue against complacency. Ample pilot studies have shown the potential benefits of public-sector ART scale-up in resource-poor settings. However, the management of ART is complex, because it entails life-long clinical monitoring and counselling to ensure desirable adherence levels. Consequently, ART programmes exert unprecedented pressure on often overburdened staff and understaffed health systems. Our findings show that, because human resource shortages are associated with lower levels of patient satisfaction, the quality of care is definitely affected by heavy workloads. Human resource shortages, combined with large numbers of patients in need of treatment, caused further extended waiting times, which were the most important source of discontent among PLWHA attending the ART assessment sites studied in the Free State. This could seriously hinder any successful ART scale-up, because several previous studies have shown that both waiting times and low patient satisfaction levels with ART-related services hinder ART adherence [44-49]. The mismatch between large patient numbers and insufficient human resources to deliver ART indicates that heavy workloads, long waiting times, and the resulting lower patient satisfaction are likely to remain problematic and even be aggravated in the years to come [3,50-52]. Collectively, our results add to the growing consensus that a long-term perspective on ART scale-up must first and foremost address the critical human resource shortages in this sphere.

Although our understanding of the complex relationships between patient satisfaction and human resource shortages in resource-constrained settings is only in its infancy, these analyses have both practical and theoretical value. From a theoretical point of view, the differences in patient satisfaction with ART-related services among districts and waves have extended current knowledge on satisfaction levels with South Africa's public-sector ART programme. They also invite further longitudinal research, to fully clarify the factors influencing patient satisfaction levels. From practical policy and management perspectives, the correlation between patient satisfaction and staffing emphasizes the need to strengthen the health system as a precondition to successful ART scale-up. In this way, this paper aims to meet the *Comprehensive Plan*'s objective of a research agenda outlining studies that will define the most effective provision of HIV and AIDS care and treatment, and guide programme implementation [2].

### Study limitations

However, there were some limitations to our study. First, the overall high levels of patient satisfaction with general services and the services performed by nurses do not necessarily warrant optimism. It has long been observed that patient satisfaction surveys consistently report high levels of satisfaction [53,54]. It has been claimed that patients' expressions of satisfaction often hide a variety of negative experiences [55]. The tendency of patients to withhold critical comment has been observed, for example, in Indonesia [56] and Lebanon [57]. The possibility of skewed high levels of patient satisfaction has also been raised in a South African study by Harrison et al. (1998) [54]. While "all" patients reported staff attitudes as "satisfying or good", the patients' responses hid "real problems": only 41% were receiving the recommended drug treatment, only 48% were given appropriate counselling, and only 37% were consulted in private.

Second and closely associated with the first limitation, the measurement of patient satisfaction with general services and with the services performed by nurses showed a skewed score distribution, with a high ceiling effect. Consequently, statistical problems arise from the lack of variability. This violates the statistical assumption of normally distributed data and limits the possibility of finding significant predictors. Research by Moret et al. (2007) showed that altering the response patterns can help to reduce the ceiling effect in future research [22,58,59].

Finally, although the second part of the analysis focused on the relationship between patient satisfaction and health staff shortages, it provided an incomplete explanation of satisfaction levels. Our findings suggest that the initial level of patient satisfaction was strongly associated with the availability of professional nurses at assessment sites. However, the differences in the evolution of patient satisfaction across districts cannot be explained only by differences in such shortages over time. Other potentially relevant structural, psychosocial, and sociobehavioral factors were not available in the dataset. The analysis also failed to include differential patient loads as a factor contributing to patient dissatisfaction. Facilities in urban/large town areas often have significantly higher patient loads than facilities located in rural/small town areas. The Free State's original staffing arrangements for the ART programme provided for three (in exceptional cases four) professional nurses at all assessment sites. Relative to both patient populations in catchment areas and national norms, professional nurse staffing for the ART programme sometimes appears generous, especially at those facilities in predominantly rural/small town districts. Consequently, high vacancy rates at rural/small town facilities do not necessarily have a negative impact on the quality of care and resulting patient satisfaction. At the other end of the spectrum, urban/large town districts often have much higher patient loads, even though nurse vacancy rates may be low. As a result, the workload of an assessment site can be high, causing lower patient satisfaction in seemingly well-staffed facilities. These possible disturbing effects of urban/large town-rural/small town differences and the resulting differential patient loads are not accounted for in our analysis.

## Conclusion

Even though South Africa still shows a relatively low coverage with ART in comparison to much poorer neighbouring countries, this study demonstrates that the overall satisfaction with ART-related services is high at assessment sites in the Free State Province. Although it is clear that the Province is capable of delivering high-quality ARV care, significant geographic and temporal differences prevail.

Human resource shortages and overburdened staff seem to threaten patient satisfaction levels at ART assessment sites in the Free State. At the start of the programme, our study evidenced significant association between patient satisfaction levels and professional nurse availability. Sufficient professional nurses seem to be a necessary condition for the successful implementation of the public-sector ART programme (at baseline), but not a sufficient one. Once the programme is up and running, and ART becomes chronic disease care, the success of the programme will probably depend on a more complex health system, providing not only biomedical but also psychological and social support. Schneider et al. (2006; 2008) already noted that it is hard to see how universal access to ART can be sustainably achieved in high-prevalence resource-limited countries without strengthened and even transformed health systems with strong community health worker programmes to address new chronic health needs [10,60].

These results suggest that further qualitative research is required to understand the factors influencing patient satisfaction with public-sector ART services in resource-limited settings.

## List of abbreviations

ART: antiretroviral treatment; ARV: antiretroviral; PLWHA: people living with HIV and AIDS; PHC: primary health-care; ANOVA: analysis of variance.

## Competing interests

The authors declare that they have no competing interests.

## Authors' contributions

EW participated in the design of the study, performed the statistical analysis, and co-wrote the manuscript. CH participated in the design of the study, gave advice in interpreting the results, and co-wrote the manuscript. DvR supervised the overall management of the longitudinal study and gave advice in interpreting the results. DvR and HM were involved in revising the article for important intellectual content. All authors read and approved the final manuscript.

## Pre-publication history

The pre-publication history for this paper can be accessed here:


